# Use of cerebrospinal fluid and serum samples impregnated on FTA^TM^ Elute filter paper for the diagnosis of infections caused by *Neisseria meningitidis*, *Streptococcus pneumoniae* and *Haemophilus influenzae*

**DOI:** 10.1371/journal.pone.0172794

**Published:** 2017-02-24

**Authors:** Lucila Okuyama Fukasawa, Maria Gisele Gonçalves, Fábio Takenori Higa, Euclides Ayres Castilho, Ana Belén Ibarz-Pavón, Claudio Tavares Sacchi

**Affiliations:** 1 Center of Immunology, Instituto Adolfo Lutz, São Paulo, São Paulo, Brazil; 2 Instituto Adolfo Lutz, São Paulo, São Paulo, Brazil; 3 Pan-American Health Organization, Washington DC, United States of America; Defense Threat Reduction Agency, UNITED STATES

## Abstract

**Background:**

The lack of information regarding the burden of acute bacterial meningitis in Latin America leads to a reduction in the estimated incidence rates of the disease, and impairs public health decisions on the use and follow-up of preventive interventions, particularly, the evaluation of existing vaccination policies. The use of the real-time PCR in diagnostic routine procedures has resulted in a substantial increase in confirmed bacterial meningitis cases. However, in resource-poor countries, these assays are only available in reference laboratories. Sample transportation to these laboratories is a critical constraint, as it requires specialized, high cost courier services. To overcome this barrier we evaluated the use of FTA^TM^ Elute filter paper cards for the conservation and processing of samples under normal environmental conditions, as they would be when transported from remote and under-equipped healthcare facilities to the reference centers. A total of 401 samples received in 2015 as part of Sao Paulo’s national surveillance for routine diagnosis were selected for this study.

**Methods:**

The sensitivity and specificity of real-time PCR were evaluated using fresh serum and cerebrospinal fluid (CSF) samples processed using our laboratory’s standard DNA extraction, and processing the same samples after being dried and stored on FTA^TM^ card, and DNA extracted following the manufacturer’s instructions.

**Results:**

The sensitivities for detection of *Neisseria meningitidis*, *Streptococcus pneumoniae*, and *Haemophilus influenzae* from CSF dried and stored on FTA^TM^ cards were 98%, 92%, and 100%, respectively, and with serum samples were 73%, 88%, and 100%, respectively. When compared to our laboratory’s standard methodology, results showed high concordance, with Kappa index ranges of 0.9877–1.00 for CSF, and 0.8004–1.00 for serum samples.

**Conclusion:**

The use of FTA^TM^ cards for CSF and serum conservation and transport represents a rapid, reliable, and cost-effective alternative that will allow obtaining valuable epidemiological information that would otherwise be lost.

## Introduction

Acute Bacterial Meningitis (ABM) is one of the most severe diseases affecting mostly children and adolescents, and remains a serious public health concern. It is estimated that up to 1.2 million cases and 135,000 deaths occur annually worldwide, and 10–20% of survivors are left with life-altering disabilities [1–3]. Three bacteria: *Streptococcus pneumoniae* (Spn), *Neisseria meningitidis* (Nm), and *Haemophilus influenzae* (Hi) are responsible for over 80% of ABM diagnosed outside the neonatal period [4]. There are currently a number of protein-polysaccharide conjugated vaccines that are effective against these pathogens when administered through childhood immunization programs [5–10], but their elevated cost presents a challenge in low and middle-income countries, where it is necessary to demonstrate their benefit to justify this investment. To this aim, the implementation of a comprehensive surveillance system encompassing epidemiological and laboratory information, and the availability of reliable diagnostic methods that allow confirmation of the etiological agent, are imperative to prove their cost-effectiveness.

In Latin America, a region that, historically, led the way on the implementation of a regional Expanded Program of Immunizations [11], all countries, with the exception of Haiti, had introduced the Hib conjugate vaccine by the end of 2006 [12], and currently 35 countries incorporate PCV10 or PCV13 in their childhood vaccination schedule. However, the lack of information regarding the burden of disease and circulating Nm strains makes the implementation of meningococcal vaccines in the region a low priority. To date, Brazil, Chile, and Argentina have included one of the available meningococcal conjugate vaccine in their schedules. These three countries have efficient surveillance systems and strong laboratory capacities that have provided the information needed to support the decision. With a few exceptions, even before vaccine implementation, the majority of countries in the region reported few to no cases of Hi, Spn and, particularly, Nm. Information available through SIREVA II, a passive laboratory-based network that collected information on isolates typed by the National Reference Laboratories from 1997 to 2012 [13–21], was valuable in guiding public health decisions regarding the use of *H*. *influenzae* type b (Hib) and pneumococcal vaccines [22–24], yet insufficient to provide evidence with regard to meningococcal vaccination [25]. Moreover, the impact of the pneumococcal vaccine in many low-income countries in Latin America is only assessed indirectly due to the difficulties in isolating the pathogen [26].

Most hospitals in Latin America have the facilities to conduct basic bacteriological analyses, but culture positivity rates are very low due to the use of antibiotics prior to sample collection, and to delay in transporting samples to the laboratory. The use of rapid diagnostic tests is often hampered by their high cost, short shelf-life, specific conservation conditions, and the skill of the laboratory technician to correctly interpret results [27]. The difficulties encountered in the isolation and characterization of bacteria directly affects the quality of data reported to epidemiological surveillance, as many cases of ABM are recorded as unknown etiology, or simply not informed due to the lack of laboratory confirmation This leads to a reduction in the estimated incidence rates of the disease, which affects both public health decision making in the use of preventive interventions in the population, and the evaluation of existing vaccination policies.

Real-time PCR assays directly from clinical samples, *i*.*e*. serum and cerebrospinal fluid (CSF), have been widely used in diagnosis of ABM caused by Hi, Spn, and Nm, and proven to be highly sensitive, specific, and reproducible even after the administration of the antibiotics [28–32]. The use of the real-time PCR in diagnostic routine procedures has contributed to substantial increase in the number of confirmed ABM cases [29–30]. However, in resource-poor countries, these assays can only be conducted in reference laboratories that have the necessary infrastructure, equipment, and trained personnel. Sample transportation to these laboratories needs to be done within a very small timeframe, and requires specific biosafety measures and preservation conditions that can only be ensured by specialized, and costly, courier services.

The use of filter paper for the long-term conservation and diagnosis of infections has proved to be a good alternative in settings with limited laboratory facilities [33–35]. Moreover, Spn and Hi have successfully been detected from dry blood and CSF samples [36–40], but there is insufficient data on the use of this technique for the detection of Nm.

In this study, we aim to evaluate the use of FTA^TM^ Elute filter paper cards for the conservation, and real-time PCR detection of Nm, Spn, and Hi from CSF and serum samples, and provide evidence of the applicability of this technique for the transport and processing of samples from remote and under-equipped healthcare facilities to the reference centers.

## Materials and methods

### Ethics statement

This study was approved by the Ethical Committee of Instituto Adolfo Lutz, by Brazil’sNational Committee for Ethics in Research (Comissão Nacional de Ética em Pesquisa, CONEP, protocol number CAAE4549613.0.0000.0059) and the Pan-American Health Organization Ethics Review Committee.

### Samples

We analyzed CSF and serum samples from suspected cases of bacterial meningitis from public health units in São Paulo city, Brazil, between March and November 2015. A total of 401 samples were received as part of Sao Paulo’s national surveillance for routine diagnosis, and were selected by order of arrival to the laboratory. Samples were anonymized and identified by a unique number before access, and no clinical or personal data were required for this study.

### DNA extraction

**Fig 1:**
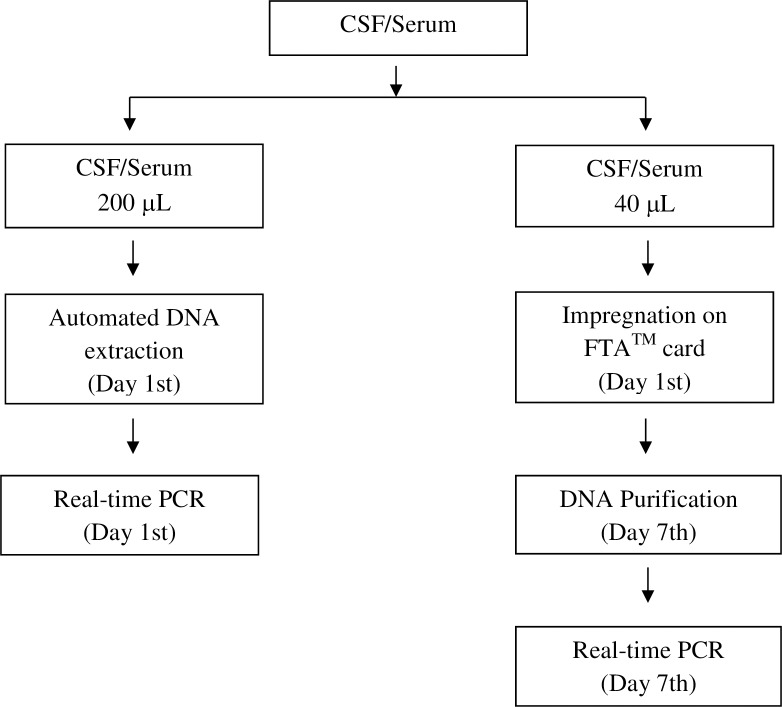
Processing algorithm of samples.

Each sample was tested simultaneously according two processing protocols (Fig 1). On day 1, 200 μL of each sample were used in automated process for DNA extraction by Roche MagNA Pure LC 2.0 equipment according to the manufacturer’s instructions. Additionally, 40 μL of each sample were impregnated on the FTA^TM^ Elute Card (GE Healthcare Life Sciences) [41], dried at room temperature for 3 hours, and stored for seven days at room temperature. On the 7^th^ day after impregnation, two 3 mm-discs were punched from the center of each card using Uni-Core^TM^ punch (GE Healthcare Lifesciences) and placed in 1.5 mL microcentrifuge tube. To prevent cross-contamination between samples, the punch was cleaned with 70% ethanol, dried with tissue paper, and punched on clean piece of filter paper three times. The punched discs were washed with 1 mL of sterile water and pulse vortexed three times for a total of 5 seconds, followed by centrifugation at 10,000 rpm for 10 seconds. Water was carefully removed using an automatic pipette, and 30 μL of molecular biology grade water were added to the tube, which was incubated in a heat block set at 98°C for 30 minutes. After pulse vortexing 60 times (one pulse/second) and centrifugation at 10,000 rpm for 30 seconds, discs were removed from the elution tube and discarded, thus obtaining the DNA.

### Real-time PCR assays

Extracted DNA from MagNA Pure were all analyzed by real-time PCR on day 1, while extracted DNA from FTA^TM^ card were all analyzed on day 7. Two different real-time PCR assays were performed for both extractions: (i) Triplex for detecting Nm (*ctrA* gene), Spn (*lytA* gene), and Hi (*hpd* gene), and (ii) Singleplex for the detection of human RNase P gene.

Assays were performed in a final volume of 25 μL using TaqMan Universal Master Mix (Applied Biosystems) with 5 μL extracted DNA and specific primers and probes described previously [29, 31]. All reactions were performed in duplicate on Applied Biosystems 7500 real-time PCR system employing the following cycling conditions: 50°C for 2 minutes, 95°C for 10 minutes followed by 45 cycles of 95°C for 15 seconds and 60°C for 1 min.

A result was considered positive when presented a cycle threshold value (Ct) less than or equal to 38 (Ct ≤ 38) and negative when Ct = 0 or Ct ≥ 39. Samples with positive results for Nm or Hi were tested in real-time PCR reactions to identify genogroups A, B, C, W, Y, and X, and genotypes a, b, c, d, e, and f using oligonucleotides described by Mothershed, and Maaroufi [42, 43], respectively.

### Data analysis

The sensitivity and specificity of real-time PCR performed on fresh samples processed by automated extraction MagNA Pure (gold standard), and the same samples dried on the FTA^TM^ card were calculated using data from 2x2 tables. The agreement between the tests was assessed using Kappa index and its 95% confidence interval (CI), and classified according to Landis and Koch (1977) on: Kappa index < 0 = no agreement; Kappa between 0 to 0.19 = low agreement; Kappa between 0.20 to 0.39 = fair agreement; Kappa between 0.40 0.59 = moderate agreement; Kappa between 0.60 to 0.79 = substantial agreement; Kappa between 0.80 to 1.00 = high concordance.

## Results

The positivity and average Cts values obtained by triplex real-time PCR using DNA extracted by automated system (MagNA Pure) or directly from the FTA^TM^ card are shown in Table 1. The test using DNA extracted from FTA^TM^ showed higher Ct values that which employed DNA extracted automatically, with a difference in Ct value of 2 to 5 cycles.

**Table 1:** Real-time PCR positivity for detection of Nm, Spn, and Hi in clinical samples received as part of Sao Paulo’s national surveillance for routine diagnosis analysis.

Sample type	N°. of positives (Ct average±SD)						N°. of negatives		Total (n = 401)
	Nm		Spn		Hi				
	Roche	FTA^TM^	Roche	FTA^TM^	Roche	FTA^TM^	Roche	FTA^TM^	
**CSF**	55 (23.6±5.2)	54 (28.3±4.6)	39 (23.4±5.8)	36 (26.2±5.2)	16 (22.7±6.2)	16 (27.6±6.1)	103	107	213
**Serum**	48 (28.9±6.3)	35 (30.6±4.8)	33 (29.1±5.8)	29 (31.8±5.7)	2 (25.5±5.5)	2 (30.5±4.5)	105	122	188

Nm, *Neisseria meningitidis*; Spn, *Streptococcus pneumoniae*; Hi, *Haemophilus influenzae*; Ct, cycle threshold; SD, standard deviation; CSF, cerebrospinal fluid. In parentheses is the average of Ct obtained in real-time PCR assay using DNA extracted by automated method (Roche MagNAPure LC 2.0) or by filter paper card FTA^TM^.

The sensitivity and specificity of triplex real-time PCR assay using purified DNA from FTA^TM^ card are shown on Tables 2 and 3. In CSF samples, we observed that the real-time PCR assay employing the FTA^TM^ card or MagNA Pure showed high correlation (Kappa = 0.9624, CI 95% 0.9259–0.9989) with a sensitivity of 96% and specificity of 100%. For detection of Nm, Spn, and Hi, the sensitivity was 98%, 92% and 100%, respectively, with high concordance (Kappa between 0.9877 and 1.00) (Table 2).

**Table 2:** Values of sensitivity, specificity and Kappa index of real-time PCR assay using purified DNA from CSF impregnated on FTA^TM^ card.

	Nm + Spn + Hi	Nm	Spn	Hi
Sensitivity	96%	98%	92%	100%
Specificity	100%	100%	100%	100%
Kappa	0.9624	0.9877	0.9515	1.0
	CI 95% [0.9259–0.9989]	CI 95% [0.9636–1.0]	CI 95% [0.8970–1.0]	CI 95% [1.0–1.0]

Nm, *Neisseria meningitidis*; Spn, *Streptococcus pneumoniae*; Hi, *Haemophilus influenzae*; CI, confidence interval; sensitivity and specificity was calculated considering the DNA extracted by the automated system Roche MagNA Pure LC 2.0 as gold standard.

**Table 3:** Values of sensitivity, specificity and Kappa index of real-time PCR assay using purified DNA from serum impregnated on FTA^TM^ card.

	Nm + Spn + Hi	Nm	Spn	Hi
Sensitivity	80%	73%	88%	100%
Specificity	100%	100%	100%	100%
Kappa	0.8126	0.8004	0.9228	1.0
	CI 95% [0.7276–0.8976]	CI 95% [0.6957–0.9051]	CI 95% [0.8480–0.9976]	CI 95% [1.0–1.0]

Nm, *Neisseria meningitidis*; Spn, *Streptococcus pneumoniae*; Hi, *Haemophilus influenzae*; CI, confidence interval; sensitivity and specificity was calculated considering the DNA extracted by the automated system Roche MagNA Pure LC 2.0 as gold standard.

In serum samples, the sensitivities were respectively 73%, 88%, and 100% for the detection of Nm, Spn, and Hi. Both methods (FTA^TM^ and MagNA Pure) showed excellent agreement of results, expressed by Kappa index between 0.8004 and 1.00 (Table 3). All CSF and serum samples processed by the FTA^TM^ card or by automated DNA extraction system were positive for the presence of human RNase P gene.

Real-time PCR assays from DNA extracted using the MagNA Pure system were able to identify 91.3% (94/103) of Nm genogroups (Nm B, n = 23; Nm C, n = 63; Nm W, n = 6; Nm Y, n = 2) and 66.7% (12/18) of Hi genotypes (Hi a, n = 5; Hi b, n = 7). Real-time PCR assays from DNA extracted using FTA^TM^ card were able to identified 87.6% (78/89) of Nm genogroups (Nm B, n = 18; Nm C, n = 54; Nm W, n = 4; Nm Y, n = 2) and 61.1% (11/18) of Hi genotypes (Hi a, n = 4; Hi b, n = 7).

## Discussion

In this study, we evaluated the use of FTA^TM^ cards in the diagnosis of infections caused by three of the major causative agents of bacterial meningitis by real-time PCR as an approach for the transport and processing of biological samples. We demonstrated that standardized routine real-time PCR assays performed on CSF and serum samples preserved dried on FTA^TM^ filter paper up to seven days maintain a level of sensitivity comparable to when performed directly on fresh biological samples. The 7 days interval for testing was chosen as it was considered to be equivalent to the maximum period for the arrival of impregnated clinical samples to the reference laboratory when sent through the regular mail service.

In the initial phase of this study, we found that there was no difference in real-time PCR positivity when we used DNA extracted from filter paper discs after 1 day or 7 days of impregnation of the sample on the card, compared to the results obtained with DNA extracted from the MagNA Pure. We also found that positivity of real-time PCR for Nm, Spn, and Hi detection was higher when we used two 3 mm-discs from FTA^TM^ card (94%) in the DNA extraction process instead of one disc (82%) (S1 Table).

Similar studies showed good performance using filter paper in molecular diagnosis of infections caused by Spn or Hi. Peltola *et al* [38] obtained a sensitivity of 92% and 70% for detection of Spn, and Hi, respectively, using impregnated CSF on filter paper and real-time PCR. Quantitative real-time PCR assays for detection of Spn and *Streptococcus suis* in CSF preserved on filter paper showed a sensitivity and specificity of 80% and 100% respectively when compared to PCR assays using DNA extracted from liquid CSF [36]. Selva *et al* [39] demonstrated that use of impregnated blood samples on filter paper resulted in 4-fold increase in Spn or Hi detection compared to standard culture. Recently, Iroh Tam *et al* [37] showed a sensitivity of 62.5% of real-time PCR assay for detection of Spn in impregnated blood samples on FTA^TM^ filter paper compared to culture.

For detection of Nm by molecular techniques using filter paper cards there is insufficient data. The study conducted by Elliot [36] analyzed the presence of Nm, Spn, and *S*. *suis* in impregnated CSF on filter paper by real-time PCR, but none was positive for Nm. In contrast, our study indicated that use of the FTA^TM^ card in the real-time PCR resulted in high levels of detection of Nm in CSF and serum. A recent study also demonstrated that Nm can be detected from nasopharyngeal swab samples impregnated in FTA^TM^ cards with sensitivity comparable to conventional culture [44]. We also showed that paper filter card may be used for molecular identification of Nm serogroups or Hi serotypes, hence providing valuable information on circulating strains, emergence of non-vaccine serotypes, and facilitating the detection of outbreaks. This is of particular relevance for meningococcal disease, for which special control measures are needed to control the spread of the infection among the patient’s close contacts, yet the administration of antimicrobial preventive treatment only occurs once the case is confirmed by the laboratory.

In our study we found that the performance of serum samples impregnated on filter paper was lower than CSF, probably due to the presence of PCR inhibitors that were not efficiently removed and/or inactivated by the chemical reagents present in the card. In addition, samples with low initial bacterial load would probably not be detected in real-time PCR assays using the filter paper, as the FTA^TM^ card employs considerably smaller amount of sample for DNA extraction (40 μL for impregnation, and two 3-mm discs for DNA extraction) compared to the automated DNA extraction system (200 μL). This was reflected in 2 to 4 units increase on the Ct values obtained in real-time PCR using DNA extracted from FTA^TM^ compared with automated DNA extraction. In the CSF samples, the average Ct value was 23.4±5.6 and 27.5±5.2 using DNA extracted from the MagNA Pure and FTA^TM^, respectively. In serum, average Ct was 28.9 ± 6.1 with the use of MagNa Pure and 31.1 ± 5.3 with use of FTA^TM^. From the 17 negative serum samples with use of the FTA^TM^ card, 13 showed high Ct values (greater than 35) in the real-time PCR with DNA extracted by MagNA Pure; the other 4 samples resulted in Ct value of 34.

The high concordance between the results of real-time PCR using MagNA Pure at Day 1 and FTA^TM^ at Day 7 indicates that the impregnated sample can be preserved at room temperature for at least one week. Other studies have shown that different types of biological materials such as CSF, whole blood and serum can be stored for long periods on filter paper for diagnostic and/or identification purposes [38, 44, 45].

Although culture is regarded as the gold standard for the diagnostic of bacterial meningitis, the sensitivity of real-time PCR has been widely demonstrated, and the technique is used in national and international reference laboratories for diagnostic and surveillance purposes in lieu of culture. For our samples, culture results were available only for 40 samples, as this information is not routinely reported from the hospitals to our laboratory. For those samples for which culture results could be obtained, 16 Nm, 17 Spn and 7 Hi, there was a 100% concordance with the results obtained using real-time PCR on impregnated samples. Moreover, Nm serogroups and Hi serotypes were also concordant.

Another limitation in our study was the low number of Hi positive samples due to the low prevalence of Hi infections in our population. However, our results demonstrate that it is possible to identify and genotype this pathogen from samples preserved on FTA^TM^ cards, thus making this a valid methodology for routine surveillance and vaccine evaluation.

The use of FTA^TM^ cards for preservation and transport of biological samples can significantly reduce the costs and facilitate the logistic of sending samples from healthcare facilities to reference laboratories. In low and middle income countries, where the etiological agent is rarely confirmed by standard bacteriology techniques, the use of real-time PCR for the detection of Nm, Spn, and Hi has the potential to significantly increase the number of confirmed cases for surveillance purposes and public health decision-making. Moreover, we demonstrated that it is possible to type both Nm and Hi. Serotying of Spn, although possible, is hampered by the amount of DNA obtained from the cards, and the fact Spn serotyping requires various sequential reactions. However, in certain situations, when there is a limited number of serotypes of interest (*e*.*g*. outbreak investigation, or vaccine failure against a specific serotype) it should be possible to obtain this information also from dried samples. For countries needing to evaluate vaccine impact, collect information to plan future interventions, or monitoring of current ones, the use of FTA^TM^ cards can overcome the limitation of poor laboratory confirmation rates. The use of a commercial system for the storage of biological samples guarantees that the product has been adequately trialed and tested for its purpose, and that it has passed the necessary quality controls to ensure consistent performance. The FTA^TM^ cards are, to our knowledge, the only commercial system available for long-term DNA storage, and are easily available throughout Latin America. Although the costs of FTA^TM^ cards paired with the use of molecular techniques can be perceived as expensive, it presents the advantage that DNA extraction and purification can be done without the use of commercial kits, which correspond to 50–60% of the final cost. Additionally, the system eliminates the need for a specialized transport, which is an important economic and logistic limiting factor when sending biological material to reference laboratories. Last but not least, the economic cost need to be measured against the long-term gains that an accurate laboratory-based surveillance system for invasive bacterial diseases in low and middle income countries could represent in terms of quality of life for vulnerable populations.

In summary, the use of FTA^TM^ cards for serum and CSF conservation and transport represents a rapid, reliable, and cost-effective alternative that will allow obtaining valuable epidemiological information that would otherwise be lost.

## Supporting information

S1 Table Real-time PCR positivity for detection of Nm, Spn, and Hi using DNA extracted by filter paper card FTA^TM^ (one or two 3 mm discs) after 1 day or 7 days of impregnation of the sample on the card. Nm, *Neisseria meningitidis*; Spn, *Streptococcus pneumoniae*; Hi, *Haemophilus influenza*e. In parentheses is the positivity of real-time PCR assay using DNA extracted by FTA^TM^ card compared to results obtained with DNA extracted from automated system Roche MagNA Pure LC 2.0.
(PDF)
