# Supplementary material for: Use of cerebrospinal fluid and serum samples impregnated on FTATM Elute filter paper for the diagnosis of infections caused by Neisseria meningitidis, Streptococcus pneumoniae and Haemophilus influenzae
Source: PLoS One. 2017 Feb 24;12(2):e0172794. doi: 10.1371/journal.pone.0172794 (PMC5325563; doi:10.1371/journal.pone.0172794)
Supplement: S1 Table — Nm, Neisseria meningitidis; Spn, Streptococcus pneumoniae; Hi, Haemophilus influenzae. In parentheses is the positivity of real-time PCR assay using DNA extracted by FTATM card compared to results obtained with DNA extracted from automated system Roche MagNA Pure LC 2.0. (PDF) [file pone.0172794.s001.pdf]

S1 Table. Real-time PCR positivity for detection of Nm, Spn, and Hi using DNA extracted by filter paper card FTA™ (one or two 3 mm discs) after 1 day or 7 days of impregnation of the sample on the card

Agents	Nº. of positives					
	1 day			7 days		
	Roche	FTA™		Roche	FTA™	
		1 disc	2 discs		1 disc	2 discs
Nm	120	99 (83%)	114 (95%)	120	97 (81%)	114 (95%)
Spn	72	59 (82%)	66 (92%)	72	59 (82%)	66 (92%)
Hi	7	6 (86%)	7 (100%)	7	6 (86%)	7 (100%)
Total	199	164 (82%)	187 (94%)	199	162 (81%)	187 (94%)

Nm, *Neisseria meningitidis*; Spn, *Streptococcus pneumoniae*; Hi, *Haemophilus influenza*e. In parentheses is the positivity of real-time PCR assay using DNA extracted by FTA™ card compared to results obtained with DNA extracted from automated system Roche MagNA Pure LC 2.0.
